# A High-Power 3P3T Cross Antenna Switch with Low Harmonic Distortion and Enhanced Isolation Using T-Type Pull-Down Path for Cellular Mobile Devices

**DOI:** 10.3390/s22145461

**Published:** 2022-07-21

**Authors:** Arash Hejazi, Reza E. Rad, S. A. Hosseini Asl, Kyung-Duk Choi, Joon-Mo Yoo, Hyungki Huh, Seokkee Kim, Yeonjae Jung, Kang-Yoon Lee

**Affiliations:** 1 Department of Electrical and Computer Engineering, Sungkyunkwan University, Suwon 16419, Korea; arash@skku.edu (A.H.); reza@skku.edu (R.E.R.); saha@skku.edu (S.A.H.A.); glyiiop@skku.edu (K.-D.C.); fiance2@g.skku.edu (J.-M.Y.); gray.huh@skku.edu (H.H.); seokkeekim@skku.edu (S.K.); yj.jung@skku.edu (Y.J.); 2 SKAIChips Co., Ltd., Suwon 16419, Korea

**Keywords:** 3P3T, harmonic distortion, insertion loss, power handling, RF switch, switching time, T-type shunt

## Abstract

This paper presents a radio frequency (RF) triple pole triple throw 3P3T cross antenna switch for cellular mobile devices. The negative biasing scheme was applied to improve the power-handling capability and linearity of the switch by increasing the maximum tolerable voltage drop across the drain and source and reverse biasing the parasitic junction diodes. To avoid signal reflection through the antenna in off-state, all the antenna ports were equipped with 50-ohm termination to provide the pull-down path. Considering the simultaneous operation of antenna ports in different switch cases, the presented T-type pull-down path demonstrated improvement of isolation by over 15 dB. Using stacked switches, the 3P3T handled the input power level of over 35 dBm. The chip was manufactured in 65 nm complementary metal oxide semiconductor (CMOS) silicon on insulator (SOI) technology with a die size of 790 × 730 µm. The proposed structure achieved insertion loss, isolation, and voltage standing wave ratio (VSWR) of less than −0.9 dB, −40 dB, and 1.6, respectively, when the input signal was 3.8 GHz. The measured results prove the implemented switch shows the second and third harmonic distortion performances of less than −60 dBm when the input power level and frequency are 25 dBm and 3.8 GHz, respectively.

## 1. Introduction

Considering the recent advancements in communication systems, mobile devices need to operate under challenging environments and different frequency bands. In a radio frequency (RF) transceiver, RF switches are widely used for band selection and duty-cycled operation between a transmitter and receiver [1,2]. As one of the critical building blocks, insertion loss directly affects the sensitivity and noise figure performance of the transceiver, and power-handling capability and linearity are other important parameters that influence the spurious free dynamic range (SFDR) of the transceiver. Thus, extensive research is in progress to meet the technical requirements under low power consumption.

Nowadays, thanks to the gate-length downscaling of silicon on insulator (SOI) technology and the provision of isolation between a device and substrate, it offers less source–drain capacitance by shrinking or removing the depletion region, which is a promising solution for high-speed applications. The linearity, power-handling capability, insertion loss (IL), return loss (RL), isolation, and switching time are the main concerns when the switch is followed by a power amplifier (PA) or low noise amplifier (LNA).

The main source of nonlinearity for a transistor in the on-state is the on-resistance (Ron) of the switch. In contrast, for a transistor in the off-state, the off-capacitance and other parasitic effects influence the nonlinearity performance of the switch [3,4]. For the transistors in the off-state, those close to antenna port and transmitter are under more stress, and in the case of applying high power levels, can be partially on, thereby providing a leakage path and decreasing the P1dB point. The body floating technique is a solution to minimize leakage and improve the IL performance of the switch. However, at higher power levels, due to the ionization effect, most carriers will be injected and stored in the body of transistors, resulting in a sudden change in threshold voltage and an increase in drain current, which is detrimental to the linearity performance. Therefore, for switches requiring higher linearity, the body is better to be contacted [5,6,7].

Adding big resistors at the gate and body of the transistors results in AC floating nodes and improves the linearity by decreasing the leakage to the control circuitry at the cost of increasing switching time [8]. The work proposed in [9] uses a negative biasing scheme to handle higher power levels by increasing the maximum tolerable voltage drop across the drain–source and reverse biasing the parasitic junction diodes. In [10] by applying the intermediate drain and source biasing method, the transistors are revered biased, and the need for a negative charge pump is relaxed. However, at higher power levels, there will be leakage to bias circuitry and those in off-state, negatively affecting the switch’s isolation performance. Another work [11] utilizes the stacking technique to distribute the voltage drop across junction capacitors to increase the power-handling capability in the off-state. A large number of stacks in a series increases the Ron of the switches and lowers the IL performance of the switch. Therefore, increasing the width of transistors is the solution to minimize the Ron.

Meanwhile, wide transistors introduce larger off-capacitance, lowering the impedance in the off-state and the isolation of the RF switch. Adding pull-down switches is a solution to improve the isolation of a switch at the cost of increasing IL and the degradation of linearity due to adding off-capacitance at the in/output port [12,13,14,15]. Considering the trade-offs between performance metrics, satisfying specifications to meet different frequency bands and standards becomes more challenging as the number of poles and throws increases.

This paper presents a high-power and linear triple pole triple throw 3P3T RF switch that employs negative biasing and a stacking technique to improve the power-handling capability and linearity of the presented architecture. To avoid signal reflection through the antenna in the off-state, all the antenna ports were equipped with a 50-ohm termination mode to provide the pull-down path. Considering the simultaneous operation of antenna ports in different switch cases and isolation problems, T-type pull-down switches were implemented to improve the isolation performance of the switch. The antenna switch handled the strong RF signal up to 35 dBm without any reliability issues. The measurement results show that the presented 3P3T achieves the second and third harmonic levels of less than −60 dBm when the input signal level is 25 dBm at 3.8 GHz. The proposed structure achieves the IL and isolation performance of less than −0.9 dB and −40 dB, respectively.

## 2. Overview of The Proposed RF-SOI Switch Unit

**Figure 1:**
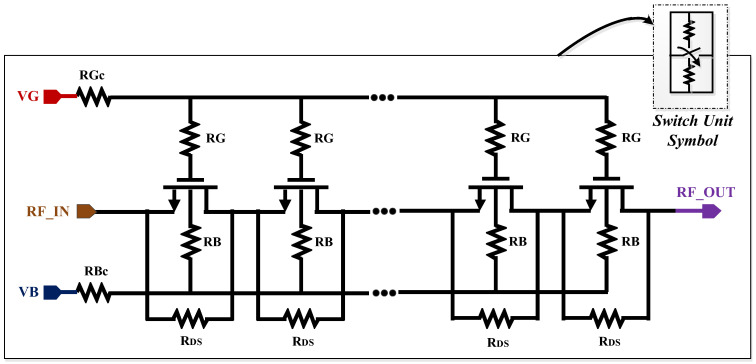
The utilized RF switch unit for series, shunt, and termination mode.

**Figure 2:**
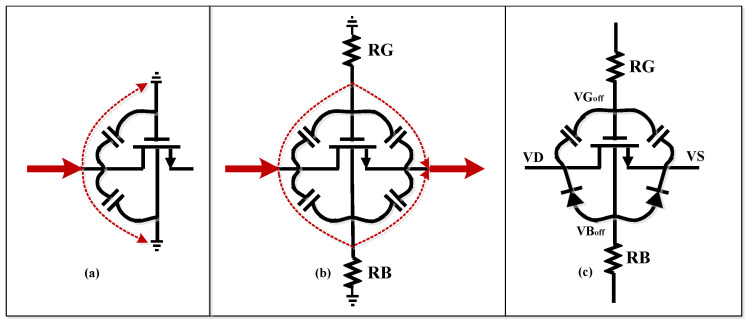
(**a**) Leakage path through junction capacitors, (**b**) blocking leakage current, (**c**) transistor model in off-state.

Figure 1 demonstrates the architecture of the proposed RF switch unit. The body and gate floating method using 150 kΩ resistors provided AC floating and high impedance nodes and reduced the RF signal leakage and the stress on the gate oxide. Figure 2a shows the signal leakage path to the gate and body of the transistors while this path was blocked with large-sized resistors at the body and gate (see Figure 2b). Thus, the resistors needed to be large enough to provide higher impedance than junction capacitors’ impedance.

In the off-state, as the input power increased due to the voltage drop between the drain and source, the drain–body and source–body junctions generated current that flowed through the added resistors and made the voltage drop across them, thereby decreasing the bias voltage and power-handling capability. Considering intrinsic design trade-off, it was desirable to keep the value of the resistor low to improve the power-handling capability and switching time, while they needed to be large enough to avoid signal leakage. In addition, to avoid voltage imbalance, a 30 kΩ resistor was connected between the drain and the source of the transistor.

As mentioned before, the main source of nonlinearity is the undesired channel formation of transistors in the off-state. Therefore, negative biasing was used to increase the maximum allowed drain–source voltage to keep the transistors in the off-state and reverse bias the junction diodes. As shown in Figure 2c, the maximum allowed drain–source voltage to keep the transistors and junction parasitic diodes in the off-state can be calculated as

(1) $$\left[VG_{off}+\left(\frac{VD+VS}{2}\right)\right]-VD<V_{th}\overset{so}{\Rightarrow }\;VSD_{max}<2\left(-VG_{off}+V_{th}\right)$$

(2) $$\left[VB_{off}+\left(\frac{VD+VS}{2}\right)\right]-VD<V_{diode}\overset{so}{\Rightarrow }\;VSD_{max}<2\left(-VB_{off}+V_{diode}\right)$$

where $VG_{off}$, $VB_{off},\;V_{th},\;\mathrm{and}\;V_{diode}$ indicate gate direct current (DC) voltage, body DC voltage, threshold voltage, and turn-off voltage of the diode, respectively. It is clear that applying negative voltage enhanced the power-handling capability of the switch.

Nine transistors were stacked to handle the input power level of 35 dBm during the off-state, and the signal swing could be divided among the stacks. It also improved the isolation performance because of a chain of series capacitors from the input to output ports. The stacked switches provided large IL. Therefore, the size of transistors in the series path was large enough (3 mm) to minimize the Ron of the switch. However, increasing the size of the switch degraded the isolation performance which was compensated by adding a shunt branch. Due to additional nonlinear capacitance, the large size of transistors in the shunt path increased the IL and degraded the linearity performance. However, considering the isolation performance and electro-static discharge (ESD) issues, the size of the switches in shunt branch was 1 mm. The size of the switches in the termination mode depended on the linearity performance when all the switches were off and only the termination mode was on. Thus, transistors with the size of 1.5 mm were chosen to satisfy the harmonic levels of less than −40 dBm in the termination mode. Considering receiving an input power level of 25 dBm in the termination mode, there would be a huge voltage swing across the resistor that could break the poly of the resistor. Therefore, two 25 Ω resistors with increased width and length were connected in a series configuration to provide the 50 Ω resistance and tolerate larger AC.

## 3. Top Block Diagram of the Proposed 3P3T Switch

**Figure 3:**
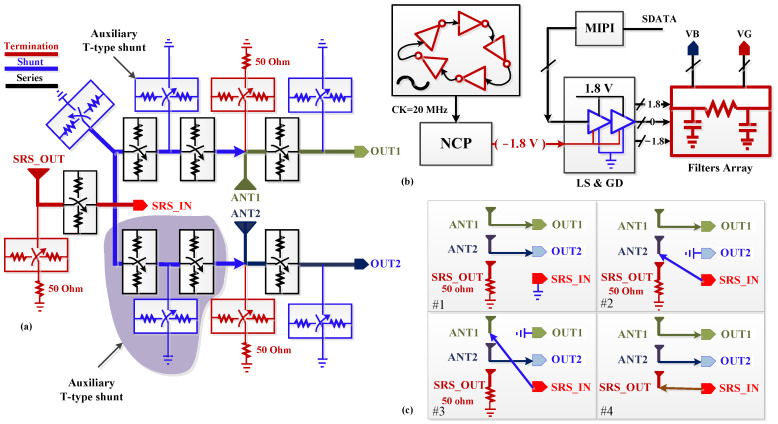
Top block diagram of the 3P3T (**a**) switches configuration, (**b**) analog part, and (**c**) switching cases.

Figure 3a illustrates the top block diagram of the proposed 3P3T. Due to multiple poles and throws and the simultaneous cross operation of them, the shunt branch could not be turned on when the target port was connected to another path. Hence, in the proposed 3P3T, to keep the isolation performance below −40 dB, auxiliary T-type pull-down switches were added to improve the isolation of the switch by breaking the series path into two parts and locating the shunt branch between them, which improved the isolation by more than 15 dB. All the antenna ports were equipped with a termination port to pull down the 3P3T when the 3P3T was off.

Figure 3b shows the controlling parts of the designed 3P3T. The voltage-controlled oscillator (VCO) using cascaded inverters in a ring [16] provided the oscillation frequency of 20 MHz which was applied to the negative charge pump (NCP). The mobile industry processor interface (MIPI) with a level shifter (LS) and gate drivers (GD) controlled the switching cases by making the gate of the transistors in the on-state 1.8 V and their body 0 V, while the gate and body of the transistors in the off-state were −1.8 V. The RC filter attenuated the RF signal and avoided signal leakage to the analog part of the 3P3T. Switching cases of the proposed 3P3T are depicted in Figure 3c. All the cases were controlled by the serial data that were received through the MIPI. As illustrated in the switching cases, the proposed structure included three antenna ports which were ANT1, ANT2, and SRS_OUT, while OUT1, OUT2, and SRS_IN were in/out ports. Therefore, this confirms the 3P3T configuration of the proposed switch.

All the antennas and ports had identical characteristics. However, to distinguish the Rx or Tx port, the OUT1 and OUT2 were specified for Rx cases that could support the high/low bands or switching to different Rx paths, while the SRS_IN was the Tx path where the PA could be connected to transfer the power through the all-antenna ports (ANT1, ANT2, and SRS_OUT).

## 4. Measurement Result

**Figure 4:**
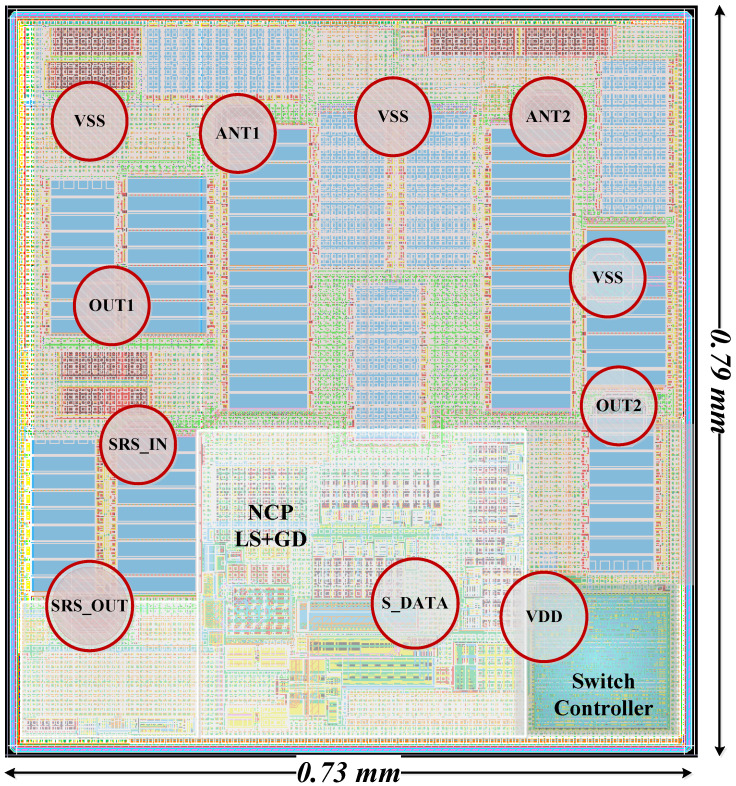
Layout pattern of the proposed 3P3T.

Figure 4 shows the layout pattern of the proposed 3P3T. The 3P3T was implemented in a 65 nm CMOS SOI process with a die size of 0.58 mm^2^. The total current consumption of the 3P3T was 110 µA from a power supply of 1.8 V.

**Figure 5:**
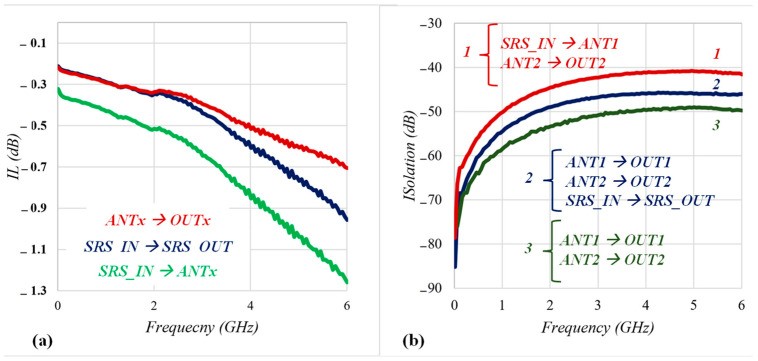
Measured result of the (**a**) IL and (**b**) isolation of the proposed 3P3T.

As depicted in Figure 5a,b, the S-parameter metrics of the presented 3P3T were measured with an Agilent E5071C network analyzer, and the frequency was swept up to 6 GHz. The 3P3T showed IL performances of less than −0.5 dB, −0.6 dB, and −0.9 dB for different switching paths. Thanks to T-type pull-down switches, it achieved less than −40 dB isolation for the worst switching case in which the Tx port (SRS_IN) was enabled and the ANT1 and ANT2 ports were connected.

**Figure 6:**
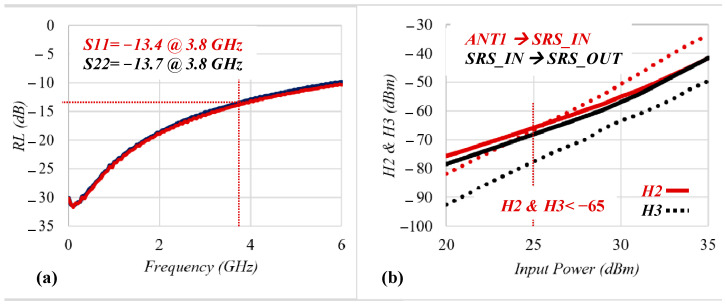
Measured results of the (**a**) RL and (**b**) harmonic distortion.

**Figure 7:**
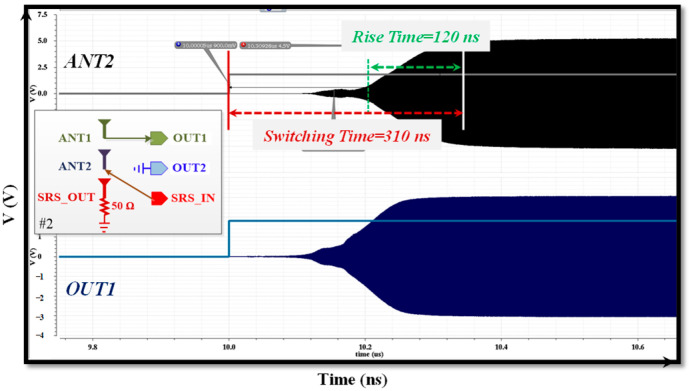
Simulation result of the switching time for switching case of 2.

Figure 6a demonstrates the RL performance of the presented architecture. It achieved RL performance of −13.4 dB and −13.7 dB at the Tx and Rx ports, respectively, which corresponded to a voltage standing wave ratio of less than 1.55 in all ports. As shown in Figure 6b, the measured power levels of the second and third harmonics at 3.8 GHz were less than −65 dBm, while for the termination mode, it was less than −40 dBm when the signal level of 25 dBm with the frequency of 902.4 MHz was applied through the antennas. Figure 7 illustrates the simulated switching time when the ANT1 to OUT1 and SRS_IN to ANT2 paths were enabled. The 3P3T achieved the switching and rise times of 310 ns and 120 ns, respectively.

Table 1 shows the comparison summary of the presented structure with recent studies and industrial products. The presented structure showed comparable IL and harmonic distortion, while thanks to using a T-type shunt path, it had superior isolation performance while improving other metrics.

## 5. Conclusions

The proposed linear 3P3T with a power-handling capability of 35 dBm employing the T-type pull-down path achieved excellent isolation without the deterioration of other performance metrics. The proposed technique enhanced the isolation performance in cross antenna switches where isolation was the primary concern. The switch was controlled via a MIPI control interface and only consumed 110 µA from a power supply of 1.8 V.

## Figures and Tables

**Table 1:** Summary of performance comparison.

Title 1	[6]	[10]	Infineon BGSX33U16	This Work
XPXT	SP7T + SP8T	SP4T	3P3T	3P3T
*f* (GHz)	0.617~2.7	2	0.699–3.8	0.699–3.8
IL (dB)	−0.34~−0.92	−0.75	−0.35~−1.5	−0.4~−0.8
Iso. (dB)	−49~−35.5	−32	−40~−37	−53~−41
RL (dB)	-	−20	−21~−13	−26.4~−13.4
H2 (dBm)	−67~−64 P_IN_ = 25 dBm	−50 P_IN_ = 33 dBm	* −72 P_IN_ = 25 dBm	** −65 P_IN_ = 25 dBm
H3 (dBm)	−72.2~−63 P_IN_ = 25 dBm	−48 P_IN_ = 33 dBm	* −72 P_IN_ = 25 dBm	−67 P_IN_ = 25 dBm
VDD (V)	1.8	2.5	1.65~3.4	1.8
Process (nm)	SOI	SOI	SOI	SOI
Area mm^2^	1.21	1.15 × 0.85	2 × 2	0.79 × 0.73

* *f* = 2.69 GHz, ** *f* = 3.8 GHz.

## Data Availability

Not applicable.
